# Leading indicators of mosquito-borne disease elimination

**DOI:** 10.1007/s12080-015-0285-5

**Published:** 2015-12-23

**Authors:** Suzanne M. O’Regan, Jonathan W. Lillie, John M. Drake

**Affiliations:** 1 Odum School of Ecology, University of Georgia, Athens, GA 30602-2202 USA; 2 National Institute for Mathematical and Biological Synthesis (NIMBioS), 1122 Volunteer Boulevard, University of Tennessee, Knoxville, TN 37996-3410 USA; 3 North Hall High School, Gainesville, GA 30506 USA

**Keywords:** Transcritical bifurcation, Critical transition, Vector-borne disease, Disease elimination, Critical slowing down, Vector-borne disease model, Malaria

## Abstract

Mosquito-borne diseases contribute significantly to the global disease burden. High-profile elimination campaigns are currently underway for many parasites, e.g., *Plasmodium* spp., the causal agent of malaria. Sustaining momentum near the end of elimination programs is often difficult to achieve and consequently quantitative tools that enable monitoring the effectiveness of elimination activities after the initial reduction of cases has occurred are needed. Documenting progress in vector-borne disease elimination is a potentially important application for the theory of critical transitions. Non-parametric approaches that are independent of model-fitting would advance infectious disease forecasting significantly. In this paper, we consider compartmental Ross-McDonald models that are slowly forced through a critical transition through gradually deployed control measures. We derive expressions for the behavior of candidate indicators, including the autocorrelation coefficient, variance, and coefficient of variation in the number of human cases during the approach to elimination. We conducted a simulation study to test the performance of each summary statistic as an early warning system of mosquito-borne disease elimination. Variance and coefficient of variation were highly predictive of elimination but autocorrelation performed poorly as an indicator in some control contexts. Our results suggest that tipping points (bifurcations) in mosquito-borne infectious disease systems may be foreshadowed by characteristic temporal patterns of disease prevalence.

## Introduction

Vector-borne diseases constitute approximately 17 % of the estimated worldwide infectious disease burden (World Health Organization 2014). Mosquito-borne infections, in particular, contribute substantially to this burden, causing millions of deaths and hundreds of millions of cases annually (World Health Organization 2013). Mosquito-borne pathogen elimination remains a high priority on the global public health agenda, with elimination campaigns underway for malaria, onchocerciasis, and lymphatic filariasis (Hopkins 2013). The introduction of insecticides such as DDT in the mid twentieth century led to the implementation of global eradication campaigns for many of the most deleterious mosquito-borne diseases, including malaria, dengue virus, and yellow fever virus. These campaigns were partially successful in that diseases were eliminated in some countries and effectively controlled in others. For example, malaria was eliminated in 79 countries between 1945 and 2010 (Chiyaka et al. 2013) and the reduction of *Aedes aegypti* led to the near elimination of dengue and yellow fever in the Americas (Gratz 1999; Gubler 1998). However, the incidence of mosquito-borne infections has resurged since the 1970s due to a nexus of factors including globalization, increased global movement of people, development of vector resistance to insecticides, changes in land use, and loss of financial support and public health infrastructure for control and elimination (Institute of Medicine (US) Forum on Microbial Threats 2008; Gubler 2010; Mackenzie et al. 2004). Sustaining political will near the end of elimination campaigns is often difficult to achieve since there may be marginal return on investment (in terms of case reductions) (Cohen et al. 2012). Government and public health agencies require markers of progress to justify the billions of dollars that are spent on interventions (Cohen et al. 2010). Quantitative evaluation tools that will encourage governments and philanthropic organizations to choose the optimal level of investment in control and elimination activities after the initial reduction of cases has occurred are needed.

Mosquito-borne disease elimination, which involves halting transmission through interventions until no parasites remain (Breman et al. 2011; Klepac et al. 2013), e.g., through vector control strategies and/or treatment of infected individuals, corresponds to a critical transition in the disease transmission system. Criticality occurs at the point where the basic reproduction number, *R*_0_, the average number of secondary infected cases arising from a single infected case in an entirely susceptible population, is equal to one. Tipping points in complex systems may be described mathematically as bifurcations if the change in the external driver variable is slow relative to the characteristic speed of the internal variables. If control efforts are implemented sufficiently slowly over time such that elimination eventually occurs, the elimination strategy causes the system to cross a bifurcation point. The critical transition may be anticipated because prior to reaching the dynamical threshold, the system gradually loses stability (“critical slowing down” Strogatz 1994; Scheffer et al. 2009). In continuous-time systems, the loss of stability is measured by the dominant eigenvalue of the linearized system. Signatures of the slow return rate to the underlying equilibrium may be detectable through summary statistics. Statistical patterns of critical slowing down may be a diagnostic of the proximity to disease elimination (e.g., O’Regan and Drake 2013). To establish how close a disease is to elimination or emergence, typically, a stochastic epidemic model (e.g., a compartmental model or a branching process model) is fitted to time series data to estimate the basic reproduction number of a disease. The disadvantage of this approach is having to specify parametric models that make strong assumptions about the form of transmission. Additionally, the fit of stochastic epidemic models to data is usually evaluated using a goodness-of-fit criterion such as statistical likelihood, but many models may yield near-identical fits. Detection of critical slowing down would circumvent the problem of model structural uncertainty and improve efficiency by making use of higher order information in time series that is not used by other approaches that focus on trends in the mean. Methods that detect the presence of critical slowing down would therefore capture key structural features of transmission that are exhibited by a large family of mosquito-borne disease models. Non-parametric approaches for emergence and elimination prediction that are independent of model-fitting and evaluation would advance infectious disease forecasting significantly.

In this paper, we use stochastic differential equations to model the gradual implementation of four control activities on mosquito-borne disease elimination: the use of bed nets that hamper the per-capita human biting rate, e.g., (Nyarango et al. 2006), indoor residual insectide spraying procedures that shorten adult mosquito lifespan (Giardina et al. 2014) or late-acting insecticides (Read et al. 2009), the administration of drugs that reduce the human infectious period (Lawpoolsri et al. 2009), and the reduction of mosquito abundance due to administration of insecticides or elimination of breeding sites through larviciding or water drainage (Goodman et al. 1999; Tusting et al. 2013). We use the models to develop and validate leading indicator summary statistics for documenting disease elimination in mosquito-borne disease systems. We focus our analysis on malaria but our findings are relevant to other mosquito-borne infections such as yellow fever. Our results indicate that critical slowing down is detectable prior to mosquito-borne disease elimination.

## Theory for elimination of vector-borne diseases

### Mean field theory of Ross-Macdonald model

We consider the Ross-Macdonald model for vector-borne disease transmission (Keeling and Rohani 2008). The Ross-Macdonald model has a long history of use as a prototypical model that encapsulates the key properties of mosquito-borne pathogen transmission (Smith et al. 2012). Human hosts and vectors (mosquitoes) are assumed to be either susceptible to disease, or infectious. Denoting the numbers of infectious human host and infectious mosquito populations by *H*(*t*) and *M*(*t*) respectively, the model is

1 $$\begin{array}{@{}rcl@{}} \dot{H} &=& \frac{k p}{N_{h}} M(N_{h}-H) - \mu H \\ \dot{M} &=&\frac{k q}{N_{h}} H(N_{m}-M)-\delta M. \end{array} $$

The population size of human hosts *N*_*h*_ is assumed to be constant and consequently, the number of susceptible human hosts *S*_*h*_ is equal to (*N*_*h*_−*H*), i.e.,

$$N_{h} = S_{h} + H.$$

Similarly, mosquito abundance *N*_*m*_ is assumed constant and the number of susceptible mosquitoes *S*_*m*_ is (*N*_*m*_−*M*), i.e.,

$$N_{m} = S_{m} + M.$$

Parameters of the model and the expression for the basic reproduction number, *R*_0_ are listed in Table 1. The model has two equilibria, the disease-free equilibrium (0, 0) and the endemic equilibrium

2 $$ (H^{\ast}, M^{\ast}) =\left( \frac{N_{h} (k^{2} N_{m} p q - N_{h} \delta \mu)}{(k q (k N_{m} p + N_{h} \mu))},  \frac{k^{2} N_{m} p q - N_{h} \delta \mu}{(k p(k q +\delta))}\right ). $$

**Fig. 1:**
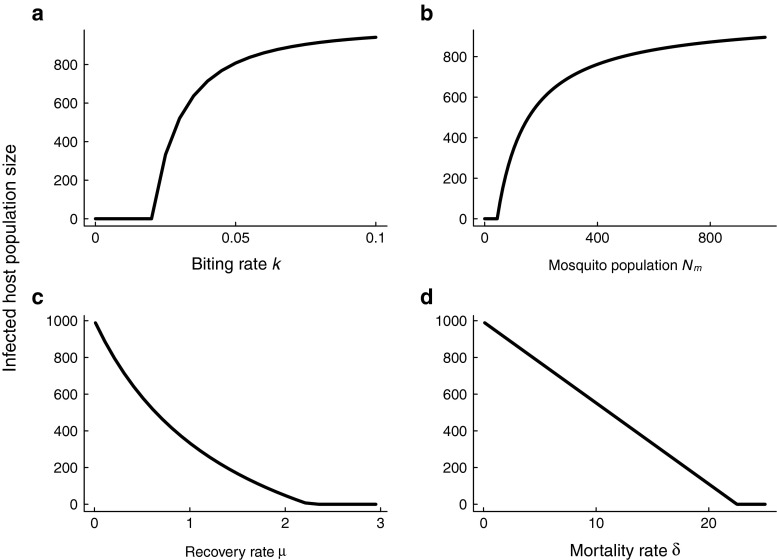
Bifurcation diagrams for the Ross-Macdonald model. The stable equilibrium branches of the transcritical bifurcation ($\epsilon \rightarrow 0$ mean field theory) as a function of each parameter affected by control activities (per-capita biting rate, mosquito population abundance, per-capita recovery rate and per-capita mortality rate) are shown. Bifurcation diagrams were plotted using the parameters given in Table 1

When *R*_0_ > 1, the endemic equilibrium is stable and the disease-free equilibrium is unstable. When *R*_0_<1, the stabilities of the equilibria are switched. Mathematically, a transcritical bifurcation, whereby the disease-free equilibrium and endemic equilibrium meet and exchange stability, occurs at the point where the basic reproduction number is equal to one (Keeling and Rohani 2008). Transcritical bifurcation diagrams corresponding to various control measures are shown in Fig. 1. Interventions due to the biting rate and mosquito abundance modify the endemic equilbrium differently than those that affect the per-capita mosquito mortality rate and per-capita human host recovery rate. The concave down trend in Figs. 1a, b suggests that successful pathogen extinction may only occur at the very end of elimination campaigns that focus on either of reduction of mosquito abundance or mosquito biting rates.

**Table 1:** Variables and parameters of the time-varying Ross-Macdonald models

Variable	Expression	Value
Number of infectious humans	*H*	
Number of infectious mosquitoes	*M*	
Per-capita human recovery rate	*μ*	0.01 day^−1^ (Smith and McKenzie 2004)
Per-capita mosquito mortality rate	*δ*	0.1 day^−1^ (Smith and McKenzie 2004)
Human population size	*N*_*h*_	1000
Per-capita mosquito biting rate	*k*	0.3 day^−1^ (Smith and McKenzie 2004)
Transmission efficiency from mosquitoes to humans	*p*	0.5 (Smith and McKenzie 2004)
Transmission efficiency from humans to mosquitoes	*q*	0.5 (Smith and McKenzie 2004)
Mosquito population size	*N*_*m*_	10000
Basic reproduction number	$R_{0} = \frac {k^{2}p q N_{m}}{\delta \mu N_{h}}$	
Time critical point is reached	*t* ^∗^	
Critical biting rate	$k^{\ast } = \sqrt {(N_{h}\delta \mu )/(pqN_{m})} $	
Critical mosquito population size	$N_{m}^{\ast } =(N_{h}\delta \mu )/(pqk^{2}) $	
Critical recovery rate	*μ* ^∗^=(*k* ^2^ *p* *q* *N*_*m*_)/(*N*_*h*_ *δ*)	
Critical mortality rate	*δ* ^∗^=(*k* ^2^ *p* *q* *N*_*m*_)/(*N*_*h*_ *μ*)	
Value of biting rate prior to application of control measures	*k*_0_	0.3 day^−1^
Rate of change in biting rate	*k*_1_	0.0001 day^−1^
Mosquito population size prior to application of control measures	*N*_*m*0_	10000
Rate of change of mosquito population size	*N*_*m*1_	1 day^−1^
Mosquito mortality rate prior to application of control measures	*δ*_0_	0.1 day^−1^
Rate of change of mosquito mortality rate	*δ*_1_	0.0025 day^−1^
Value of human recovery rate prior to application of control measures	*μ*_0_	0.01 day^−1^
Rate of change of recovery rate	*μ*_1_	0.001 day^−1^
Time-varying biting rate	$k(t) = \left \{\begin {array}{ll} k_{0} - k_{1} t, &t < t^{\ast } \\ k^{\ast } &t \geq t^{\ast } \end {array}\right .$	*t* ^∗^=2800 days
Time-varying mosquito population size	$N_{m}(t) = \left \{\begin {array}{ll} N_{m0} - N_{m1} t, &t < t^{\ast } \\ N_{m}^{\ast } &t \geq t^{\ast } \end {array}\right .$	*t* ^∗^=9956 days
Time-varying mortality rate	$\delta (t) = \left \{\begin {array}{ll} \delta _{0} + \delta _{1} t, &t < t^{\ast } \\ \delta ^{\ast } &t \geq t^{\ast } \end {array}\right .$	*t* ^∗^=8960 days
Time-varying recovery rate	$\mu (t) = \left \{\begin {array}{ll} \mu _{0} + \mu _{1} t, &t < t^{\ast } \\ \mu ^{\ast } &t \geq t^{\ast } \end {array}\right .$	*t* ^∗^=2240 days
Environmental noise strength	*σ*	0.05

**Fig. 2:**
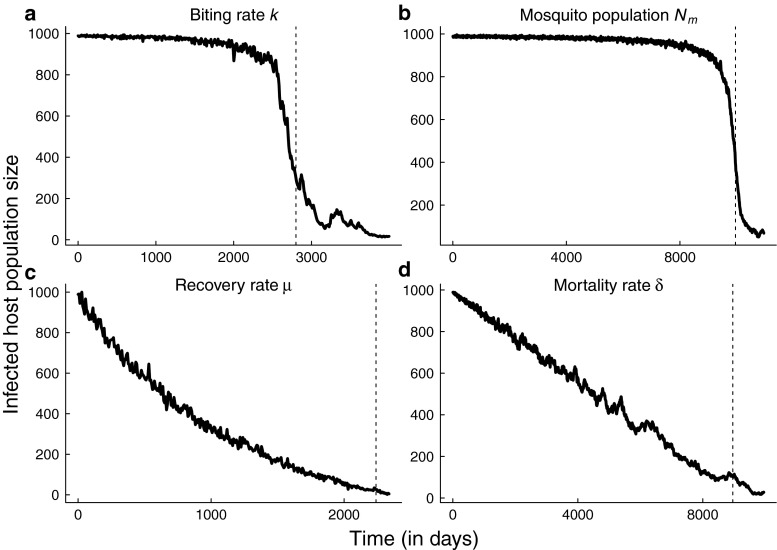
Stochastic simulations of the Ross-Mcdonald system approaching elimination due to slow declines in **a** per-capita biting rate, **b** mosquito population size, and slow increases in **c** per-capita recovery rate **d** per-capita mosquito mortality rate. The *dashed vertical line* indicates the critical threshold for extinction of the pathogen in the deterministic system. The time to parasite extinction is longer in the fast-slow stochastic systems than in the corresponding deterministic systems

Control strategies for mosquito-borne diseases include bed net use, application of insecticides or modification of breeding habitat, and prompt administration of drug treatment. Control activities rolled out over long time scales relative to disease transmission cause disease prevalence to slowly decline, eventually pushing the transmission system over the tipping point to elimination (Fig. 2). For example, the rate of bed net use among children across four districts in Kenya steadily increased from 7 % in 2004–2005 to 67 % in 2006–2007 (Noor et al. 2007). To represent the implementation of control strategies over long time frames, we assume model parameters affected by control measures may be written as time-varying functions (Table 1). For example, if the per-capita biting rate *k* of mosquitoes decreases at rate *k*_1_ due to gradual increases in bed net usage, we write down the Ross-Macdonald equations as a fast-slow system (e.g., Kuehn 2011),

3 $$\begin{array}{@{}rcl@{}} \dot{H} &=& \frac{k p}{N_{H}} M(N_{H}-H) - \mu H \end{array} $$

4 $$\begin{array}{@{}rcl@{}} \dot{M} &=&\frac{k q}{N_{H}} H(N_{M}-M)-\delta M,  \\ \dot{k} &=& \epsilon f (t,  H,  M),  \end{array} $$

where *𝜖* is a parameter that denotes the speed of evolution of the biting rate (assumed to be gradual, i.e., 0<*𝜖*<<1) and the function *f* describes the change in biting rate. The biting rate evolves over time to its critical value *k*^∗^ (achieved at *R*_0_=1 at time *t* = *t*^∗^) according to

$$k(t) = \left\{\begin{array}{ll} k_{0} - k_{1} t,  &t < t^{\ast}\\ k^{\ast} &t \geq t^{\ast} \end{array}\right. $$

and consequently, the change in biting rate is slow relative to the time scale of transmission since $|\dot {k}| = k_{1} << k^{\ast } < k_{0}$. We use similar systems of equations to model the reduction of the duration of the infectious period (1/*μ*(*t*)), the reduction of mosquito population numbers (*N*_*m*_(*t*)), and the increase of per-capita mosquito mortality rate (*δ*(*t*)) over time *t* through control applications (Table 1).

#### Stochastic description of Ross-Macdonald model

We assume population fluctuations are due to demographic and environmental stochasticity. All individuals of each type (humans, mosquitoes) have identical attributes, in that per-capita transmission and recovery rates for all human hosts are the same for all individuals and per-capita transmission and mortality rates are the same for all mosquito hosts. The human population size is assumed constant and equal to *N*_*h*_ (constant and equal to *N*_*m*_ for the mosquito population). Individuals may transition from being susceptible to being infectious. Table 2 shows the transition probability fluxes into and out of the infectious populations (*H*, *M*). We use the diffusion approximation outlined in Allen (2003) to derive stochastic differential equations that incorporate demographic stochasticity and time-varying control measures. In Appendix 1, we provide the derivation of the equations.

**Table 2:** Transition probability fluxes for Ross-Macdonald model. Numbers of infectious humans and populations are denoted by *X*=(*H*, *M*)^*T*^. The vector Δ*X*=(Δ*X* ^*i*^)=(*H*(*t*+Δ*t*)−*H*(*t*), *M*(*t*+Δ*t*)−*M*(*t*))^*T*^ denotes change in state, *i*=1, 2, …, 5

Event *i*	Change in population sizes	(Δ*X* ^*i*^)	Transition probability *p*_*i*_
Infection of susceptible humans	$(H, M)\rightarrow (H+1, M)$	$ \left (\begin {array}{l} 1 \\ 0 \end {array}\right ) $	$p_{1} = \frac {k(t) p M(N_{h}-H)}{N_{h}} {\Delta } t$
Recovery of infectious humans	$(H, M) \rightarrow (H-1, M)$	$\left (\begin {array}{l} -1 \\ 0 \end {array}\right )$	*p*_2_ = *μ*(*t*)*H*Δ*t*
Infection of susceptible mosquitoes	$(H, M) \rightarrow (H, M+1)$	$\left (\begin {array}{l} 0 \\ 1 \end {array}\right )$	$ p_{3} = \frac {k(t) q H(N_{m}(t)-M)}{N_{h}} {\Delta } t$
Death of infectious mosquitoes	$(H, M) \rightarrow (H, M-1)$	$\left (\begin {array}{l} 0 \\ -1 \end {array}\right )$	*p*_4_ = *δ*(*t*)*M*Δ*t*
No change	$(H, M) \rightarrow (H, M)$	$\left (\begin {array}{l} 0 \\ 0 \end {array}\right )$	$ 1 - \displaystyle \sum \limits _{i=1}^{4} p_{i} $

Transitions are presented in their most general form by expressing parameters that may be influenced by control measures as functions of time

Environmental variations, e.g., in temperature, are important determinants of mosquito dynamics, driving fluctuations in mosquito abundance and influencing mosquito life histories and behaviors, e.g., (Rogers et al. 2002; Paaijmans et al. 2009; Paaijmans et al. 2013). Furthermore, there may be variability in individual adherence to drug treatment (e.g., Langton et al, 2015). We modify the equations obtained from the diffusion approximation to include the effects of environmental stochasticity in control activities (see Appendix 1 for details). We assume the stochastic process obtained from the diffusion approximation is perturbed by an additional environmental noise term that scales with the mean level of the rate impacted by a control activity at time *t*. Specifically, in a small time interval Δ*t*, we assume each time-varying control measure *g*(*t*) is subject to environmental stochasticity as follows,

5 $$ g(t) {\Delta} t = (g_{0} + g_{1} t) {\Delta} t + \sigma \eta \sqrt{\Delta t},  $$

where *η* is a normal random variate with mean zero and unit variance and *σ* denotes the strength of the environmental noise. Equation 5 replaces the appropriate parameter in the drift term obtained through the diffusion approximation (A.1 in Appendix 1), to represent an environmental perturbation to each process. For example, assuming per-capita biting rate is gradually reduced through control efforts, the change in the human host and mosquito populations in a small time interval [*t*, *t*+Δ*t*) is

6 $$\begin{array}{@{}rcl@{}} H(t+{\Delta} t) &=& H(t) + \left( \frac{k(t) p}{N_{h}} M(t)(N_{h} - H(t))\right ){\Delta} t \\ &+&\sqrt{\frac{k (t)p}{N_{h}} M(t)(N_{h} - H(t)) + \mu H(t)} \eta_{1} \sqrt{\Delta t} \\ &+& \sigma \frac{M(t) p (N_{h} - H(t))}{N_{h}} \eta_{3} \sqrt{\Delta t}\\ M(t\, +\, {\Delta} t)&\!\!\!\, =\, \!& \!\!M(t) \, +\,  \left( \!\frac{k(t) q}{N_{h}} H(t)(N_{m} \, -\,  M(t)) \, -\,  \delta M(t)\!\right)\!{\Delta} t \\ &+&\sqrt{\frac{k(t) q}{N_{h}} H(t)(N_{m} - M(t)) + \delta M(t)} \eta_{2} \sqrt{\Delta t} \\ &+& \sigma \frac{H(t) q (N_{m}-M(t))} {N_{h}}\eta_{3} \sqrt{\Delta t},  \end{array} $$

where *η*_*i*_ are normally distributed random variables with mean zero and variance of unity. Letting Δ*t*→0, and assuming existence and uniqueness of the stochastic integral (Allen 2003), then $\eta _{i} \sqrt {\Delta t} \rightarrow 0$ and the system of equations converges in the mean square sense to a system of Ito stochastic differential equations,

7 $$\begin{array}{@{}rcl@{}} dH &=& \left( \frac{k(t) p}{N_{h}} M(N_{h} - H) - \mu H\right) dt\\ &&+\sqrt{\frac{k (t)p}{N_{h}} M(N_{h} - H) + \mu H} dW_{1} + \sigma \frac{M p (N_{h} - H)}{N_{h}} dW_{3} \\ dM&=& (\frac{k(t) q}{N_{h}} H(N_{m} - M) - \delta M) dt\\ &&+\sqrt{\frac{k(t) q}{N_{h}} H(N_{m} - M) + \delta M}dW_{2} + \sigma \frac{H q (N_{m}-M)} {N_{h}} dW_{3}.\\ \end{array} $$

The systems of stochastic differential equations in Table 3 corresponding to each control activity were derived in the same way (Appendix 1). Simulations of each model approaching elimination are illustrated in Fig. 2.

**Table 3:** Time-varying Ross-Macdonald equations with demographic and environmental stochasticity

Biting rate	$\begin {array}{llll} dH = (\frac {k(t) p}{N_{h}} M(N_{h} - H) - \mu H) dt +\sqrt {\frac {k (t)p}{N_{h}} M(N_{h} - H) + \mu H} dW_{1} + \sigma \frac {M p (N_{h} - H)}{N_{h}} dW_{3} \end {array}$
	$\begin {array}{llll} dM = (\frac {k(t) q}{N_{h}} H(N_{m} - M) - \delta M) dt+\sqrt {\frac {k(t) q}{N_{h}} H(N_{m} - M) + \delta M}dW_{2} + \sigma \frac {H q (N_{m}-M)} {N_{h}} dW_{3} \end {array}$
Mosquito population size	$\begin {array}{llll} dH = (\frac {k p}{N_{h}} M(N_{h} - H) - \mu H) dt +\sqrt {\frac {k p}{N_{h}} M(N_{h} - H) + \mu H} dW_{1} \end {array}$
	$\begin {array}{lll} dM = (\frac {k q}{N_{h}} H(N_{m}(t) - M) - \delta M) dt+\sqrt {\frac {k q}{N_{h}} H(N_{m} - M) + \delta M}dW_{2} + \sigma \frac {k H q} {N_{h}} dW_{3} \end {array}$
Recovery rate	$\begin {array}{llll} dH = (\frac {k p}{N_{h}} M(N_{h} - H) - \mu (t) H) dt +\sqrt {\frac {k p}{N_{h}} M(N_{h} - H) + \mu (t) H} dW_{1} + \sigma H dW_{3} \end {array}$
	$\begin {array}{lll} dM = (\frac {k q}{N_{h}} H(N_{m} - M) - \delta M) dt+\sqrt {\frac {k q}{N_{h}} H(N_{m} - M) + \delta M}dW_{2} \end {array}$
Mortality rate	$\begin {array}{llll} dH = (\frac {k p}{N_{h}} M(N_{h} - H) - \mu H) dt +\sqrt {\frac {k p}{N_{h}} M(N_{h} - H) + \mu H} dW_{1} \end {array}$
	$\begin {array}{llll} dM = (\frac {k q}{N_{h}} H(N_{m} - M) - \delta (t) M) dt+\sqrt {\frac {k q}{N_{h}} H(N_{m} - M) + \delta (t) M}dW_{2} + \sigma M dW_{3} \end {array}$

Five hundred simulations of each set of equations were performed. Terms under square roots represent the *G*_11_ and *G*_22_ entries in the diffusion matrix *G*(*t*)(A.5), whereas terms that scale with the environmental noise strength *σ* are the *G*_*i*3_ entries

### Leading indicators of elimination

To calculate leading indicators of disease elimination, we need to analyze the properties of the time-varying Ross-Macdonald system, in the neighborhood of the equilibrium point that would be present in the *𝜖*→0 limit (e.g., O’Regan and Drake 2013; Kuehn 2011). In the stochastic system, the equilibrium is a quasi-stationary state. Quantifying the behavior of the deviations from the equilibrium point can be achieved by deriving a locally linear approximate description of the probability distribution that is the solution of the forward Kolmogorov equation (A.6). Since the Ross-Macdonald system does not exhibit long transients and the time-varying functions represent non-stationary processes that are changing gradually through time, linearization of the drift and diffusion terms of the equations in Table 3 gives reasonable information for the qualitative behavior of fluctuations about the quasi-stationary state.

Solutions of the linearized forward Kolmogorov equation (A.7) have the same probability distribution as the system of the stochastic differential equations (A.8). In Appendix 1, we apply the Fourier transform to these equations to derive the power spectrum of the fluctuations in human cases. Finally, the leading indicator statistics (variance, autocorrelation coefficient, and the coefficient of variation) are obtained from integration of the power spectrum. The expressions for the leading indicators are found in Table 4 and are expressed in terms of the eigenvalues of the Jacobian matrix of the system linearized at the endemic equilibrium. In Appendix 1, we show that the endemic equilibrium of system (1) is always a stable node if the parameters of the model are positive. Consequently, we present the expressions for the statistics in terms of the real and distinct dominant and subdominant eigenvalues, *λ*_1_ and *λ*_2_, respectively. Changes in these summary statistics as the bifurcation point is approached are indicators of critical slowing down.

**Table 4:** Analytical expressions for quasi-stationary statistics about the endemic infectious human quasi-steady state *H* ^∗^ expressed in terms of the eigenvalues

Power spectrum *S*_*I*_(*ω*)	$\frac {2(\alpha _{H}+D_{11} \omega ^{2})}{(\omega ^{2}-\lambda _{1}\lambda _{2})^{2}+(\lambda _{1}+ \lambda _{2})^{2} \omega ^{2}}$
Autocorrelation	$\displaystyle \frac {1}{\pi }{\int }_{0}^{\infty } S_{I}(\omega ) \cos (\omega \tau ) d\omega $
Variance *σ* ^2^	$\frac {\alpha _{H} +\lambda _{1}\lambda _{2} D_{11}}{2 \lambda _{1}\lambda _{2} (-\lambda _{1}- \lambda _{2})}$
Coefficient of variation	$\frac {(\alpha _{H} +\lambda _{1}\lambda _{2} D_{11})^{\frac {1}{2}}}{\sqrt {2 \lambda _{1}\lambda _{2} (-\lambda _{1}- \lambda _{2})} H^{\ast }}$

The equilibrium of the Ross-Macdonald model, (*H* ^∗^, *M* ^∗^), is a stable node (Appendix 1) and thus the Jacobian matrix has two real, negative, distinct eigenvalues, *λ*_1_ and *λ*_2_. Variables for each model are described in Tables 1,  5, and 6. The expressions for the power spectrum are multiplied by 2 because they are evaluated over the frequency domain $[0,  \infty )$. No closed-form expression for the lag- *τ* autocorrelation is known and so it must be evaluated numerically

**Table 5:** Variables substitutions for the stochastic differential equations for the fluctuations about the endemic equilibrium (2) at time *t* (A.12)

Variable	Expression
*H* ^∗^	$\frac {N_{h} (k(t)^{2} N_{m}(t) p q - N_{h} \delta (t)\mu (t))}{k(t) q (k(t) N_{m}(t) p + N_{h} \mu (t))}$
*M* ^∗^	$\frac {k(t)^{2} N_{m}(t) p q - N_{h} \delta (t) \mu (t)}{k(t) p(k(t) q +\delta (t))}$
*a*_11_	−*k*(*t*)*p* *M* ^∗^/*N*_*h*_−*μ*(*t*)
*a*_12_	*k*(*t*)*p*(*N*_*h*_−*H* ^∗^)/*N*_*h*_
*a*_21_	*k*(*t*)*q*(*N*_*m*_(*t*)−*M* ^∗^)/*N*_*h*_
*a*_22_	−*k*(*t*)*q* *H* ^∗^/*N*_*h*_−*δ*(*t*)
*d*	*a*_11_ *a*_22_−*a*_12_ *a*_21_
*T*	−*a*_11_−*a*_22_
*α*_*H*_	$a_{22}^{2} D_{11}+a_{12}^{2} D_{22}-2D_{12}a_{12}a_{22}$

If the statistics were evaluated about the trajectory of the fast-slow system, then *H* ^∗^ and *M* ^∗^ in the table below are replaced with *H*(*t*) and *M*(*t*) respectively (e.g., Fig. 4). The expressions for the *D*_*ij*_ coefficients are in Table 6. For fluctuations about an endemic equilibrium, the value of control parameters *k*(*t*), *N*_*m*_(*t*), *μ*(*t*) and *δ*(*t*) are constant

**Table 6:** Variables substitutions for terms of the variance-covariance matrix of time-varying Ross-Macdonald models with demographic and environmental noise

Coefficient	Biting rate	Recovery rate
*D*_11_	$\frac {k(t) p}{N_{h}} M^{\ast }(N_{h} - H^{\ast }) + \mu (t) H^{\ast } + (\sigma /Nh)^{2} p^{2} (M^{\ast })^{2} (N_{h} - H^{\ast })^{2} $	$ \frac {k p}{N_{h}} M^{\ast }(N_{h} - H^{\ast }) + \mu (t)H^{\ast } + (\sigma H^{\ast })^{2}$
*D*_12_	(*σ*/*N* *h*)^2^ *p* *q* *M* ^∗^ *H* ^∗^(*N*_*h*_−*H* ^∗^)(*N*_*m*_−*M* ^∗^)	0
*D*_21_	(*σ*/*N* *h*)^2^ *p* *q* *M* ^∗^ *H* ^∗^(*N*_*h*_−*H* ^∗^)(*N*_*m*_−*M* ^∗^)	0
*D*_22_	$\frac {k(t) q}{N_{h}}H^{\ast }(N_{m} - M^{\ast }) + \delta M^{\ast }+(\sigma /Nh)^{2}q^{2}(H^{\ast })^{2}(N_{m} - M^{\ast })^{2}$	$\frac {k q}{N_{h}}H^{\ast }(N_{m} - M^{\ast }) + \delta M^{\ast }$
Coefficient	Mosquito population size	Mortality rate
*D*_11_	$\frac {k p}{N_{h}}M^{\ast }(N_{h} - H^{\ast }) + \mu H^{\ast }$	$\frac {k p}{N_{h}} M^{\ast }(N_{h} - H^{\ast }) + \mu H^{\ast }$
*D*_12_	0	0
*D*_21_	0	0
*D*_22_	$\frac {k q}{N_{h}}H^{\ast }(N_{m}(t) - M^{\ast }) + \delta M^{\ast } +\sigma ^{2} \frac {k q}{N_{h}}(H^{\ast })^{2}$	$\frac {k q}{N_{h}}H^{\ast }(N_{m} - M^{\ast }) + \delta (t) M^{\ast }+(\sigma M^{\ast })^{2}$

If the statistics were evaluated about the trajectory of the fast-slow system, then *H* ^∗^ and *M* ^∗^ in the table below are replaced with *H*(*t*) and *M*(*t*), respectively (e.g., Fig. 4). For fluctuations about an endemic equilibrium, the value of control parameters *k*(*t*), *N*_*m*_(*t*), *μ*(*t*) and *δ*(*t*) are constant

### Simulations

The preceding sections present an analytical theory of leading indicators of elimination for stochastic time-varying Ross-Macdonald models. To investigate the results of this theory for a particular parameter set, we calculated leading indicators of disease elimination in infectious human hosts using the models in Table 3, assuming that (a) the mean number of infectious individuals in the *𝜖*→0 limit is given by the deterministic endemic equilibrium (*H*^∗^, *M*^∗^) (2) or (b) by assuming it is given by the current state (*H*(*t*), *M*(*t*) of the system approaching elimination. We selected parameters consistent with malaria (Smith and McKenzie 2004) (Table 1).

To test the robustness of the theoretical predictions in Table 4, we simulated the approach to elimination 500 times for each of the models in Table 3. For each control scenario, the infectious time series approaching elimination were sampled at weekly intervals in each simulation, assuming perfect detection (no underreporting). The transcritical bifurcation in these scenarios was approached over a long time frame (e.g., 2800 days (400 weeks) when *k*_1_=1/10, 000 day^−1^) in the fast-slow Ross-Macdonald model approaching elimination. The length of the time series in each simulation set depended on how long it took to reach the critical threshold *R*_0_=1 in each model (400 weeks in the changing biting rate model, 1280 weeks in the changing mosquito mortality model, 320 weeks in the changing recovery rate model, and 1424 weeks in the changing mosquito abundance model, respectively).

### Analysis over a moving window

Our simulation study evaluates the performance of summary statistics (lag-1 autocorrelation, variance, and coefficient of variation) as an early warning system. Here, we describe an algorithm to process input time series data that is independent of a specific model. To investigate the robustness of the changes in the early warning theoretical predictions over a moving window, i.e., as they would be used in online analysis of surveillance data, we used Gaussian filtering to remove the influence of the slowly varying trend (Dakos et al. 2008). We fitted a Gaussian kernel smoothing function with a fixed bandwidth across the infectious human host time series up to the time that the transcritical bifurcation was predicted (*t*^∗^). We obtained the residuals by subtracting the fit from each time series. We calculated the lag-1 autocorrelation, variance, and coefficient of variation of the residuals over a moving window half the length of each time series. We calculated the lag-1 autocorrelation coefficient of each replicate using the acf function in *R*. The coefficient of variation was found by calculating the mean and standard deviation of each infectious replicate. The median and 95 % prediction intervals for each of the statistics were calculated over the 500 replicates of each model. The prediction intervals were calculated using the quantile function in *R*. To quantify the association between time and the statistic for each replicate, we used Kendall’s correlation coefficient *τ*, a non-parametric statistic of association between two quantities that has values between 1 (positive correlation) and -1 (negative correlation). By repeating the calculation for each realization of leading indicators, we generated distributions of the temporal correlation *τ* for all of the simulation sets approaching criticality.

To assess leading indicator performance, we adapted the method described in Boettiger and Hastings (2012) to distinguish between statistics obtained from systems approaching elimination from those calculated from quasi-stationary systems. Distributions of Kendall’s *τ* were additionally generated using the statistics obtained from realizations of quasi-stationary epidemic systems (null models) that were processed using the procedure outlined above. That is, the null models have the same environmental noise structure as the test models (the systems of stochastic differential equations in Table 3) but assume *k*(*t*) = *k*_0_, *N*_*m*_(*t*) = *N*_*m*0_, *δ*(*t*) = *δ*_0_ and *μ*(*t*) = *μ*_0_ in each model respectively. Null models were initialized from the deterministic equilibrium calculated from the values of *k*_0_, *N*_*m*0_, *μ*_0_, and *δ*_0_ in Table 1 and were simulated for the same length of time as required for the transition to be approached in the test models (systems approaching elimination).

From the distributions of Kendall’s *τ* obtained from null and test model realizations, we calculated receiver-operating characteristic (ROC) curves. The receiver-operating curve plots diagnostic sensitivity (true positive rate, y-axis) as a function of one minus diagnostic specificity (false positive rate). The false positive rate is the integral of the distribution of the correlation coefficent under the quasi-stationary system, to the right of a threshold line, and the true positive rate is the integral of the distribution of the correlation coefficent under the system approaching elimination. These rates make up the curve. If curves lie on or near a line with a constant slope of unity, this would imply the distributions overlap completely, and they cannot be used to distinguish between quasi-stationary systems from those approaching criticality (Boettiger and Hastings 2012; O’Regan and Drake 2013). The magnitude of departure from this line is summarized by the area under the curve, which reaches its maximum at one. If the area under the curve is close to one, then there is near perfect detection of sensitivity.

### Underreporting

To assess the effects of underreporting on leading indicator performance, we binomially sampled each time series in each set of stationary and supercritical intervention simulations with rates of detection ranging from 20 to 90 %. We calculated the ROC curves and reported the area under the curve for each simulation set.

## Results

### Predictions for mean behavior of leading indicators

**Fig. 3:**
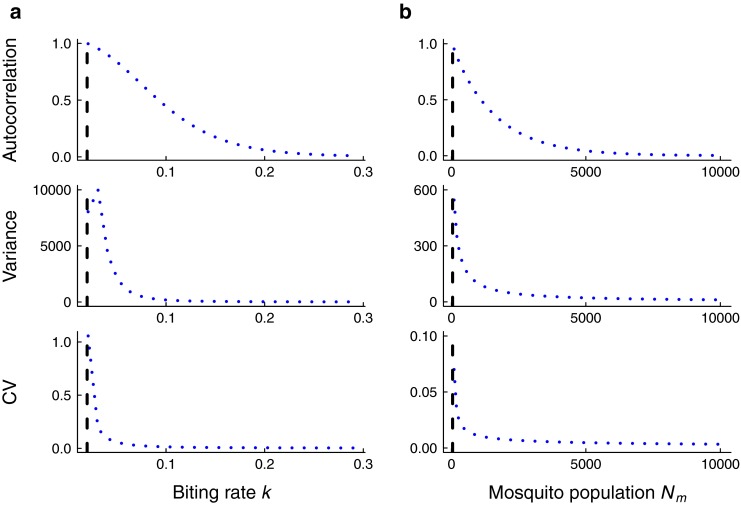
To obtain predictions for how the summary statistics behave as elimination is approached, mean leading indicators were calculated numerically using parameter values relevant for malaria (Table 1). The *vertical dashed line* in each figure indicates the threshold mosquito population abundance and threshold per-capita biting rate at *R*_0_=1 respectively. **a** Lag-1 autocorrelation and coefficient of variation are predicted to increase as control measures impacting per-capita biting rate are applied but variance becomes non-monotonic close to the critical point. **b** Lag-1 autocorrelation, variance, and coefficient of variation are predicted to increase as control actitivies affecting mosquito population abundance are applied

**Fig. 4:**
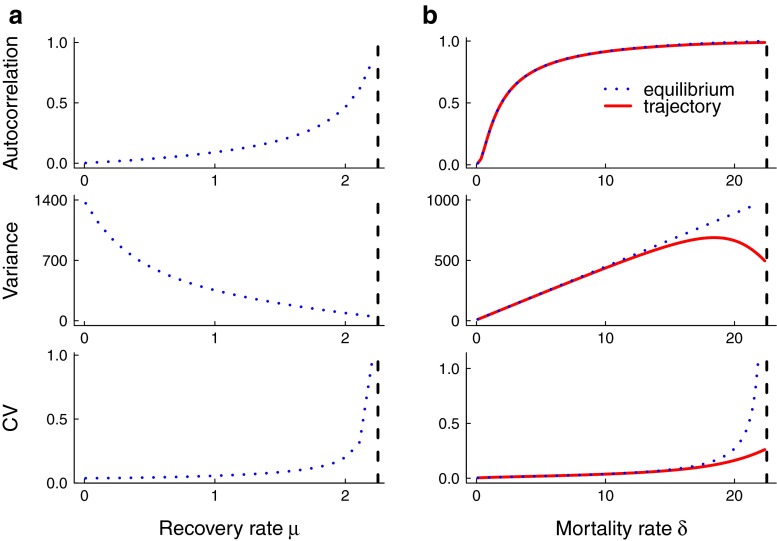
To obtain predictions for how the summary statistics behave as elimination is approached, mean leading indicators were calculated numerically using parameter values relevant for malaria (Table 1). The *vertical dashed line* in each figure indicates the threshold per-capita recovery rate and threshold per-capita mosquito mortality rate at *R*_0_=1 respectively. **a** Lag-1 autocorrelation and coefficient of variation are predicted to increase as control measures that affect the human infectious period are applied but variance is predicted to decrease. **b** Lag-1 autocorrelation, variance and coefficient of variation are predicted to increase as the per-capita mosquito mortality rate increases due to control activities. Here, we compare the statistics evaluated at the equilibrium (*H* ^∗^, *M* ^∗^) and along the fast-slow trajectory (*H*(*t*), *M*(*t*)). We note that the variance is non-monotonic if evaluated along the trajectory, but there is agreement further from the threshold

Leading indicators of mosquito-borne parasite elimination exhibit systematic changes as the critical point is approached. Figure 3 shows the theoretical predictions for the lag-1 autocorrelation, variance, and coefficient of variation when the per-capita biting rate and mosquito abundance respectively gradually decline over time. In both cases, the lag-1 autocorrelation, variance, and coefficient of variation are predicted to increase as the system nears criticality. However, the increase in variance is not sustained as the biting rate of mosquitoes is lowered. Figure 4 shows the predictions as the per-capita recovery and mosquito mortality rates respectively are increased. The lag-1 autocorrelation and the coefficient of variation are both predicted to increase as control measures are applied but the variance is predicted to decline as the infectious period shortens (Fig. 4a). Variance is predicted to increase as the per-capita mosquito mortality rate increases but the variance is a non-monotonic function of mortality rate (Fig. 4b). In all cases in Figs. 3 and 4, the theoretical predictions were calculated using the eigenvalues arising from linearizing each system about its equilibrium because the equilibrium was sufficiently close to the trajectory of the fast-slow systems in Table 1. Only for the mosquito mortality rate was there a difference between the theoretical equilibrium and the observed trajectory (Fig. 4b).

### Simulation study results

**Fig. 5:**
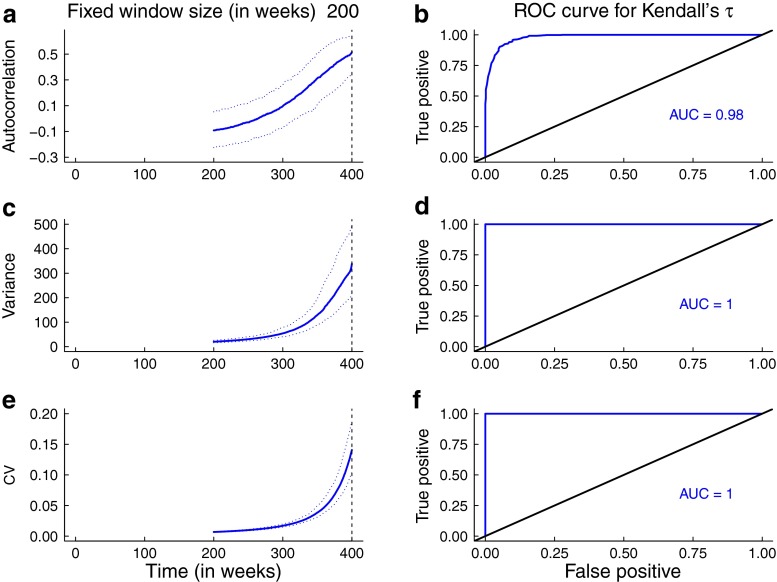
Simulation study results arising from arising from reduction in per-capita biting rate. Note that the value of the biting rate is continuously changing over the 200-week window, at a rate of $k_{1} = 1/10, \! 000~\text {day}^{-1}$. Panels **a**, **c**, and **e** show the median statistics (thick lines) and 95 % prediction intervals (*shaded regions*). The *dashed vertical line* marks the time of the transcritical bifurcation. The trends in the median statistics agree with the mean theoretical predictions (Fig. 3a). Panels **b**, **d**, and **f** show the results of the ROC analysis. The AUCs are high, indicating it is possible to distinguish between the stationary system and one slowly approaching elimination. A bandwidth of 80 weeks was selected for Gaussian filtering

**Fig. 6:**
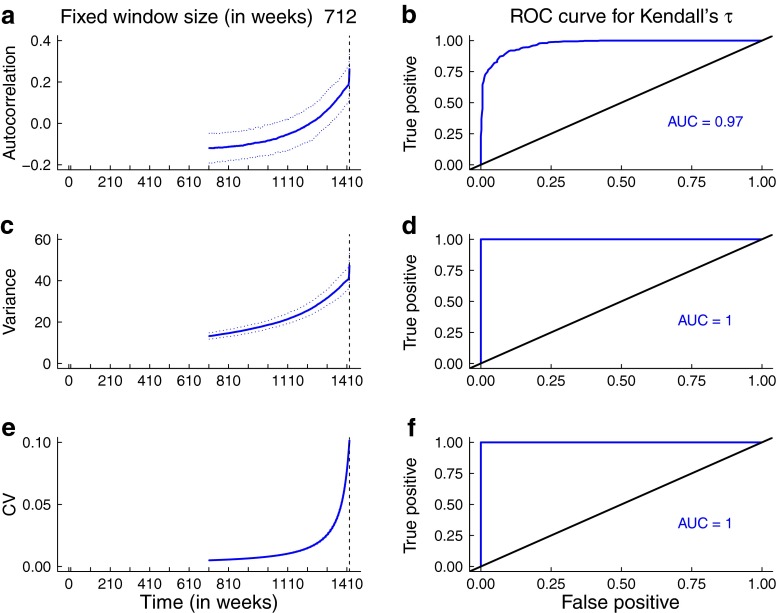
Simulation study results arising from reduction in mosquito population size. Note that the value of mosquito abundance is continuously changing over the 712-week window, at a rate of $N_{m1}~=~1~\text {day}^{-1}$. Panels **a**, **c**, and **e** show the median statistics (*thick lines*) and 95 % prediction intervals (*shaded regions*). The *dashed vertical line* marks the time of the transcritical bifurcation. Theoretical predictions for the trends in each summary statistic are robust over a moving window. The AUCs are high, indicating it is possible to distinguish between the stationary system and one slowly approaching elimination. A bandwidth of 80 weeks was selected for Gaussian filtering

**Fig. 7:**
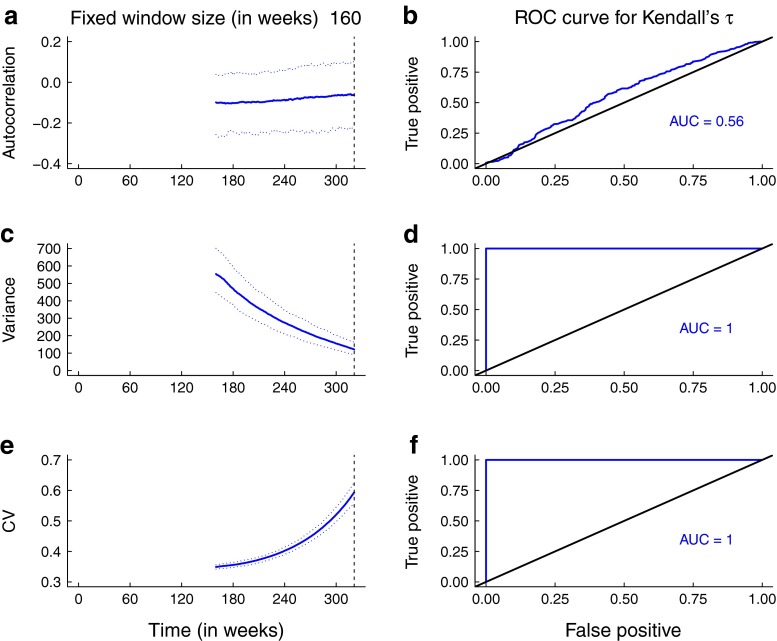
Simulation study results obtained from reduction in human infectious period. Note that the value of the recovery rate is continuously changing over the 160-week window, at a rate of $\mu _{1}~=~1/1000~\text { day}^{-1}$. Panels **a**, **c**, and **e** show the median statistics (*thick lines*) and 95 % prediction intervals (*shaded regions*). The *dashed vertical line* marks the time of the transcritical bifurcation. Theoretical predictions for trends in variance and coefficient of variation are robust over a moving window. Panels **b**, **c**, and **f** show the performance of the statistics, assessed through ROC analysis. The AUCs are high, indicating it is possible to distinguish between the stationary system and one slowly approaching elimination but the AUC value for the autocorrelation indicates it is less accurate in distinguishing between the stable system and one approaching criticality. A bandwidth of 80 weeks was selected for Gaussian filtering

**Fig. 8:**
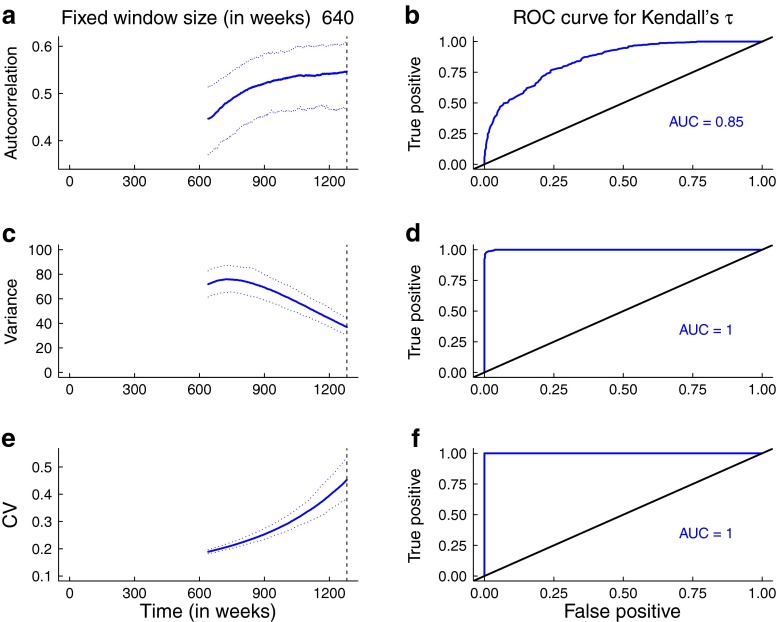
Simulation study results obtained from reduction in per-capita mosquito mortality rate. Note that the value of the mortality rate is continuously changing over the 640-week window, at a rate of $\delta _{1}~=~0.0025~\text {day}^{-1}$. Panels **a**, **c**, and **e** show the median statistics (*thick lines*) and 95 % prediction intervals (*shaded regions*). The *dashed vertical line* marks the time of the transcritical bifurcation. Theoretical predictions evaluated about the trajectory (*red lines* in Fig. 4b) are robust over a moving window. Panels **b**, **c**, and **f** show the performance of the statistics, assessed through ROC analysis. The AUCs are high, indicating it is possible to distinguish between the stationary system and one slowly approaching elimination. A bandwidth of 80 weeks was selected for Gaussian filtering

The predictions for the statistics obtained from the simulations are broadly consistent with the theoretical predictions for the mean statistics. In Figs. 5 and 6, the trends in the median and 95 % prediction intervals for the summary statistics (autocorrelation, variance, coefficient of variation) as per-capita biting rate and mosquito abundance respectively agree with the mean theoretical predictions (Fig. 3). Additionally, the areas under the ROC curves are close to 1, indicating that it is possible to distinguish between quasi-stationary systems and systems approaching elimination due to control activities that impact mosquito behavior and abundance. However, predictions from simulations over a moving window obtained from reducing the infectious period of human hosts and the lifespan of mosquitoes respectively are not as robust (Figs. 7 and 8). As the infectious period is shortened, theoretical predictions for variance and coefficient of variation are robust over a moving window, but the AUC value for lag-1 autocorrelation indicates that autocorrelation is a less accurate indicator of elimination in this scenario. The less accurate performance of autocorrelation as an indicator is also seen as mosquito lifespan is shortened due to control measures. Variance is predicted to decline as elimination is approached, matching the theoretical prediction along the trajectory (Fig. 4b).

### Imperfect detection results

**Fig. 9:**
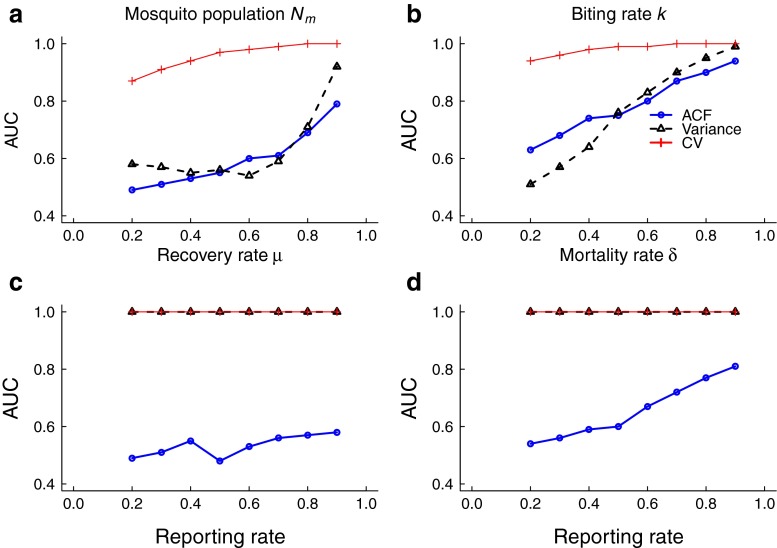
Effects of imperfect detection on leading indicator performance for each intervention: **a** Changing mosquito abundance, **b** changing biting rate, **c** changing recovery rate, and **d** changing mosquito mortality rate. Each time series was binomially sampled with detection rates ranging from 20 to 90 %. The area under the curve (AUC) is graphed as a function of detection rate. The coefficient of variation (CV) appears to be the most robust indicator to underreporting

As expected, the accuracy of the leading indicators, measured by AUC, generally increases with detection rate (Fig. 9). The coefficient of variation appears to be the most robust indicator under the effects of observation error while the performance of variance and autocorrelation generally appear to be sensitive to the effects of underreporting.

## Discussion

Detecting the onset of critical transitions in infectious disease systems such as the emergence of novel pathogens and the elimination of disease would be of tremendous value for public health. The goal of our study was to develop a theory of leading indicators for vector-borne infectious disease transmission systems that follow the assumptions of the Ross-Macdonald model and that are forced through a critical transition through gradual increases in control efforts. Our main results include analytical expressions for summary statistics for a family of time-varying Ross-Macdonald models approaching elimination (Table 4). Numerical calculations assuming time-varying control activities indicate that trends in the observable statistics are discernible. Testing the robustness of these predictions via a simulation study suggests that critical slowing down is detectable in human-host fluctuations of Ross-Macdonald type mosquito-borne disease systems. These results are in broad agreement with the findings that variance, autocorrelation, and coefficient of variation were predictive of disease elimination, in supercritical SIR and SIS compartmental models with time-varying vaccination uptake and transmission rates respectively (O’Regan and Drake 2013).

**Fig. 10:**
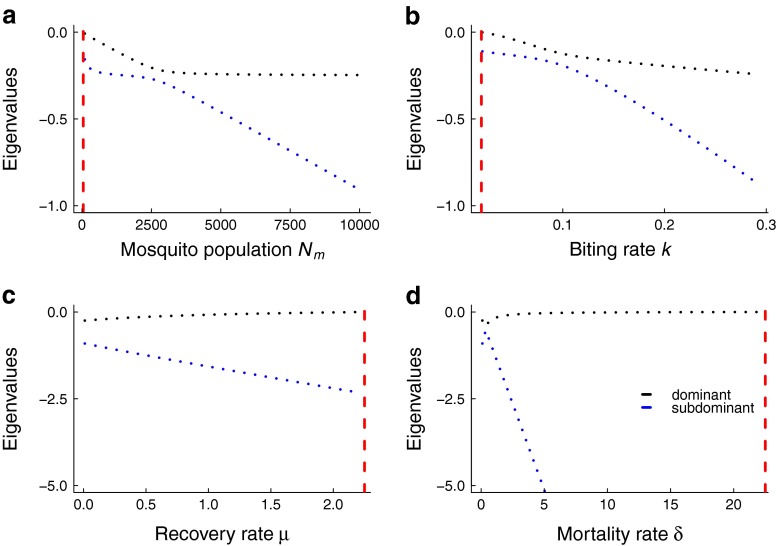
Eigenvalues obtained from linearization about the stable node equilibrium corresponding to each control activity. The *red dashed vertical line* corresponds to the critical value of each parameter where *R*_0_=1. Parameter values relevant for malaria were used (Table 1)

The simulation study suggests that variance and coefficient of variation perform well as leading indicators of elimination in all control contexts, but autocorrelation performs poorly if control strategies affect the removal rates of either human host or mosquito populations, manifested as reduction in human infectious period or mosquito lifespan respectively. In contrast, if control activities influence the transmission rate, either by reducing mosquito abundance or per-capita biting rate, all of the statistics, including autocorrelation, perform strongly. The poor performance of autocorrelation is somewhat surprising, as it has been suggested that autocorrelation may be the most robust indicator of critical transitions, at least for fold bifurcations (Dakos et al. 2012). One hypothesis for this finding is how the eigenvalues of the system change as criticality is approached. For the numerical parameters considered here, time-varying control measures that result in reduction of transmission result in eigenvalues that decline with control (albeit at different rates), whereas measures that reduce the removal rate can lead to non-monotonic eigenvalues, e.g., the subdominant eigenvalue exhibiting increases in magnitude, rather than decreasing in magnitude, as might be expected (Fig. 10). More research is needed to investigate how dynamical changes in eigenvalues impact the summary statistic predictions generally. Interestingly, the autocorrelation appears to respond earlier than the coefficient of variation to interventions (Figs. 5,  6,  7, and 8). The speed of the response of leading indicators to gradual parameter changes, and determining the factors most important in driving statistical patterns of critical slowing down, are subjects of ongoing research.

The ROC procedure we describe here is not appropriate for measuring leading indicator statistics in time series of case reports. What is observed in practice is a single realization (case report time series) that may lie far from the mean model prediction due to stochastic fluctuations. Rather, we use the theory to calculate the theoretical values of the mean leading indicator summary statistics and compare changes in them as the critical point is approached with changes in median statistics from 500 stochastic simulations over a moving window (“Simulations” and “Analysis over a moving window” sections; and “Results” section). Changes in summary statistics as the critical point is approached are signatures of critical slowing down. Our simulation study tests the performance of each summary statistic as an early warning system, which we define as an information processing system that returns a binary signal at each time in the simulation.

Our online processing algorithm assumes that the input data is the number of infectious individuals at time *t*, but in practice, data would consist of incidence time series (the number of new cases at time *t*). Incidence data will closely agree with the number of currently infected individuals if the incidence time series has the same resolution as the duration of the infectious period (Ferrari et al. 2005). If the system is quasi-stationary or moving towards elimination sufficiently slowly, then the number of infectious individuals at a time equal to the infectious period should equal the number of new cases. However, this issue becomes complicated for diseases such as malaria where case reports in elimination settings may only include very sick individuals that seek treatment and not those who are infected but are not sick enough to seek treatment. Moreover, our results in Fig. 9 suggest that the effects of imperfect detection are not uniform across interventions. Development of theory for detection of critical slowing down accounting for underreporting and the presence of unobserved processes is needed.

The Ross-MacDonald model has long been used as a strategic model for understanding mosquito-borne infectious disease (Smith et al. 2012), but has not been studied under the realistic scenario of a slow environmental forcing. Actual elimination requires the growth of institutions (like hospitals, central government, land conversion like ditching, and mosquito control networks) that are slow to develop and need to be maintained until elimination is reached. While many control campaigns could be considered “pulse” interventions, in that the intervention is deployed as quickly as possible, the models considered here encompass “press” strategies (interventions that are maintained until elimination is reached). Our approach ignores integrated control measures that impact multiple components of vectorial capacity simultaneously, e.g., insecticides targeted on adult mosquitoes that affect mosquito abundance and adult longevity. However, it would be straightforward to derive the theoretical predictions for any such model, simply by appropriate modification of the formulas for the eigenvalues. A limitation of our time-varying models is that they do not include the extrinsic incubation period (EIP), to which transmission can be sensitive to variation. Including a delay in the form of an exposed mosquito class could be important for anticipating elimination if evolution-proof control activities that affect mosquito lifespan are implemented since sustained transmission between hosts and vectors requires the EIP to be less than adult lifespan. Additionally, our simple model of mosquito-borne disease elimination does not include many realistic aspects of mosquito-borne dynamics including waiting time distributions (Smith and McKenzie 2004), temperature dependence in the gonotrophic cycle and mosquito development time (Paaijmans et al. 2013; Read et al. 2009), spatial location of hosts and vectors, and spatial heterogeneity in adult and larval mosquito habitats (Hollingsworth et al. 2014; Perkins et al. 2013; Reiner et al. 2013). Future tactical models examining elimination dynamics should include these complexities but since most models of vector-borne diseases are of the Ross-Macdonald type (Reiner et al. 2013), it is reasonable to develop theoretical predictions for summary statistics using the existing Ross-Macdonald theory, combined with simple assumptions of control activities applied to malaria.

In conclusion, to our knowledge, this study constitutes the first theory for non-parametrically anticipating elimination of mosquito-borne diseases. Our analysis of Ross-Macdonald models parameterized for malaria shows that critical slowing down is detectable in fluctuations in human cases for mosquito-borne parasites approaching elimination, suggesting that disease elimination may be anticipated even in the absence of a detailed understanding of underlying mechanisms. Our work suggests that online algorithms for detecting changes in leading indicators may be achievable, possibly aiding sustainment of the gains made by elimination programs.

## A Appendix

**Derivation of stochastic differential equations used in simulations**

### Demographic stochasticity

To formulate a continuous stochastic host-vector model, we begin by writing down a discrete stochastic process by listing each event that can occur and its corresponding transition probability (Table 2). Letting *X*(*t*)=(*X*_1_, *X*_2_)^*T*^ = (*H*(*t*), *M*(*t*))^*T*^ and applying the diffusion approximation, we calculate the expectation vector and the covariance matrix for the change in state vector Δ*X* = (Δ*X*^1^, Δ*X*^2^)^*T*^ = (Δ*H*, Δ*M*)^*T*^. From the central limit theorem, we assume Δ*X*=(Δ*X*^*i*^) is normally distributed for sufficiently large Δ*X* and for small Δ*t*. Neglecting higher order terms in Δ*t*, up to leading order, the expectation vector is

A.1 $$ E({\Delta} X) \, =\, \! \sum\limits_{i=1}^{5} \!p_{i} {\Delta} X^{i} \, =\,  \left( \!\begin{array}{l} \frac{k(t) p}{N_{h}} M(N_{h} - H) - \mu(t) H \\ \frac{k(t) q}{N_{h}} H(N_{m}(t) - M) - \delta(t) M \end{array}\!\right)\! {\Delta} t \!= \mathbf{\!\mu} {\Delta} t. $$

Denoting the transpose of a matrix by a superscript *T*, the covariance matrix is given by

$$\begin{array}{@{}rcl@{}} E(({\Delta} X) ({\Delta} X)^{T}) &=& \displaystyle {\sum}_{i=1}^{5} p_{i} ({\Delta} X^{i})({\Delta} X^{i})^{T}\\[-2pt] &=&p_{1} \left( \begin{array}{ll}1 & 0 \\ 0 & 0 \end{array}\right) +p_{2} \left( \begin{array}{ll}1 & 0 \\ 0 & 0 \end{array}\right)+p_{3} \left( \begin{array}{ll}0 & 0 \\ 0 & 1 \end{array}\right)+p_{4} \left( \begin{array}{ll}0 & 0 \\ 0 & 1 \end{array}\right)\\[-2pt] &=& \left( \begin{array}{cc} \frac{k(t) p}{N_{h}} M(N_{h} - H(t)) + \mu(t) H(t) & 0 \\ 0 & \frac{k(t) q}{N_{h}} H(t)(N_{m}(t) - M(t)) + \delta(t) M(t) \end{array}\right) {\Delta} t = \mathbf{M}(t) {\Delta} t. \end{array} $$

Letting $\mathbf {B}(X(t),  t)) = \sqrt {\mathbf {M}(X(t),  t)}$, the probability distribution of solutions to the discrete-valued continuous process satisfies the forward Kolmogorov equation (Allen 2003),

A.2 $$\begin{array}{@{}rcl@{}}</p><p class="noindent"> \frac{\partial {\Pi}}{\partial t} &=& - \sum\limits_{i=1}^{2}\frac{ \partial} {\partial X_{i}}(\mu_{i}(X(t), t) {\Pi}(X(t),  t))\\ &&+ \frac{1}{2} \sum\limits_{i=1}^{2}\sum\limits_{j=1}^{2}\frac{ \partial} {\partial X_{i} X_{j}}(B_{ij}(X(t), t) {\Pi} (X(t),  t)). \end{array} $$

The probability distribution π(*X*(*t*), *t*) is identical to the distribution of solutions to the system of stochastic differential equations (Allen 2003),

A.3 $$\begin{array}{@{}rcl@{}} dH &=& \mu_{1}(X(t),  t)) dt + B_{11}(X(t),  t)) dW_{1}\\ dM &=& \mu_{2}(X(t),  t)) dt + B_{22}(X(t),  t)) dW_{2} \end{array} $$

where *d**W*_*i*_ are independent Wiener processes with mean zero and variance *dt* respectively.

### Environmental stochasticity

Environmental variation is included into the model (A.3) by adding an environmental perturbation to system (A.3) that scales with the rate that is altered by a particular control activity. Thus, we modify the Eq. A.3 yielding,

A.4 $$\begin{array}{@{}rcl@{}} dH &=& \mu_{1}(X(t),  t)) dt + G_{11}(X(t),  t)) dW_{1} + G_{13}(X(t),  t)) dW_{3}\\ dM &=& \mu_{2}(X(t),  t)) dt + G_{22}(X(t),  t)) dW_{2} + G_{23}(X(t),  t)) dW_{3},  \end{array} $$

where the diffusion matrix *G*(*t*) is given by

A.5 $$ \mathbf{G(t)} = \left( \begin{array}{ccc} B_{11}(X(t),  t)) & 0 & G_{13}(X(t),  t)) \\ 0 & B_{22}(X(t),  t)) & G_{23}(X(t),  t)) \end{array}\right),  $$

and *d**W*_3_ is a Wiener process. The entries *G*_13_ and *G*_23_ in *G*(*t*) represent environmental noise terms. Each model in Table 3 describing a time-varying control measure and environmental stochasticity may be written in the general form given by Eq. A.4. For example, in the system of stochastic differential equations with time-varying biting rate in Table 3, $G_{11} = \sqrt {\frac {k (t)p}{N_{h}} M(N_{h} - H) + \mu H}$, *G*_12_ = *G*_21_=0, $G_{22} = \sqrt {\frac {k (t)q}{N_{h}} H(N_{m} - M) + \delta M}$, *G*_13_ = *σ**M**p*(*N*_*h*_−*H*)/*N*_*h*_ and *G*_23_ = *σ**H**q*(*N*_*m*_−*M*)/*N*_*h*_. Solutions of system (A.4) have the same probability distribution *P*(*X*(*t*), *t*) as solutions of the forward Kolmogorov equation (Allen et al. 2008) given by

A.6 $$\begin{array}{@{}rcl@{}} \frac{\partial P(X(t),  t)}{\partial t} &=& -\sum\limits_{i=1}^{2}\frac{ \partial} {\partial X_{i}}(\mu_{i}(X(t),  t)) P(X(t),  t))\\ &&+ \frac{1}{2} \sum\limits_{i=1}^{2} \sum\limits_{j=1}^{2}\frac{ \partial} {\partial X_{i} X_{j}} [P (X(t),  t)\\ &&\sum\limits_{l=1}^{3}(G_{il}(X(t),  t)G_{jl}(X(t),  t)) )]. \end{array} $$

Finally, the dimension of system (A.4) may be reduced by noting that the solutions of the system (A.4) have the same probability distribution as the following system of stochastic differential equations (Allen et al. 2008),

A.7 $$\begin{array}{@{}rcl@{}} dH &=& \mu_{1}(X(t), t) dt +\sqrt{ V_{11}(X(t),  t))} dW_{1}^{*} + \sqrt{V_{12}(X(t),  t))} dW_{2}^{*} \\ dM &= & \mu_{2}(X(t), t) dt +\sqrt{ V_{21}(X(t),  t))} dW_{1}^{*} + \sqrt{V_{22}(X(t),  t))} dW_{2}^{*},  \end{array} $$

where $dW_{i}^{*}$ are independent Wiener processes and **V**(*t*) denotes the covariance matrix,

$$\mathbf{V}(X(t),  t) = \left( \begin{array}{cc} B_{11}(X(t),  t)) + G_{13}(X(t),  t))^{2} & G_{13}(X(t),  t)) G_{23}(X(t),  t)) \\ G_{13}(X(t),  t)) G_{23}(X(t),  t)) & B_{22}(X(t),  t)) + G_{23}(X(t),  t))^{2} \end{array}\right). $$

The covariance matrix **V**(*X*(*t*), *t*)) is related to **G**(*X*(*t*), *t*)) by **V**(*X*(*t*), *t*)) = **G**(*X*(*t*), *t*))**G**(*X*(*t*), *t*))^*T*^. System (A.7) is associated with the following forward Kolmogorov equation,

A.8 $$\begin{array}{@{}rcl@{}} \frac{\partial P(X(t),  t)}{\partial t} \!\!&=&\! \, -\,  \sum\limits_{i=1}^{2}\frac{ \partial} {\partial X_{i}}(\mu_{i}(X(t), t) P (X(t),  t))\\ &&+\frac{1}{2}\sum\limits_{i=1}^{2}\sum\limits_{j=1}^{2}\frac{ \partial} {\partial X_{i} X_{j}}(V_{ij}(X(t),  t) P (X(t),  t)). \end{array} $$

### Linearization

To derive early warning signals of disease elimination, we seek parametric expressions for the fluctuations about the endemic equilibrium. Since the Ross-Macdonald system does not exhibit long transients and the time-varying functions representing non-stationary processes are changing gradually through time, linearization gives reasonable information for the behavior of fluctuations. We linearize the functions *μ*_*i*_(*H*(*t*), *M*(*t*), *t*) and *V*_*i**j*_(*H*(*t*), *M*(*t*), *t*) about the endemic equilibrium (*H*^∗^, *M*^∗^) at time *t*. Letting *x*=(*x*_1_, *x*_2_)=(*H*(*t*)−*H*^∗^, *M*(*t*)−*M*^∗^) denote a perturbation from the endemic equilibrium at time *t* and retaining leading order terms from Taylor expansions of each function about (*H*^∗^, *M*^∗^), we obtain

$$\begin{array}{@{}rcl@{}} \mu_{i} \!\!&\approx&\!\! \mu_{i}(H^{\ast},  M^{\ast},  t)\, +\, \frac{\partial \mu_{i}(H^{\ast},  M^{\ast},  t)}{\partial H} x_{1} \, +\, \frac{\partial \mu_{i}(H^{\ast},  M^{\ast},  t)}{\partial M} x_{2} \, +\,  {\dots} \\ \!\!&\approx&\!\! 0 \, +\,  \displaystyle \sum\limits_{j=1}^{2} a_{ij} x_{j} \dots \end{array} $$

where *a*_*i**j*_ denote the partial derivatives of *μ*_*i*_ and

$$\begin{array}{@{}rcl@{}} V_{ij} &\approx& V_{ij}(H^{\ast},  M^{\ast},  t)+ {\dots} \\ &=& D_{ij},  \end{array} $$

where *D*_*i**j*_ denotes the evaluation of *V*_*i**j*_ at the endemic equilibrium. The forward Kolmogorov equation for joint probability distribution of the deviations from equilibrium *p*(*x*, *t*) is

A.9 $$\begin{array}{@{}rcl@{}} \frac{\partial p(x, t)}{\partial t} &=& - {\sum}_{i=1}^{2}\frac{ \partial} {\partial x_{i}}p (x,  t)\left( {\sum}_{j=1}^{2} a_{ij} x_{j} \right)\\ &&+\frac{1}{2}{\sum}_{i=1}^{2}{\sum}_{j=1}^{2}\frac{ \partial} {\partial x_{i} x_{j}}(D_{ij}(x) p (x,  t)). \end{array} $$

The entries of the linearization matrix **A** and the corresponding covariance matrix **D** for each time-varying control measure are expressed in Tables 5 and 6 respectively.

### Derivation of leading indicators of elimination

We note that any function *x*(*t*) defined in the time interval −*T*/2≤*t*≤*T*/2 may be written in terms of its Fourier transform $ \tilde {x}(\omega )$,

A.10 $$ x(t) = \frac{1} {2 \pi} {\int}_{-\infty}^{\infty} \tilde{x}(\omega) \exp (i \omega t) d\omega $$

where *ω* denotes angular frequency. Roughly (A.10) describes a superposition of amplitudes of all the angular frequency components of the signal *x*(*t*) and thus Fourier transformation is a convenient means for discerning its underlying frequencies (Nisbet and Gurney 1982). The Fourier transform of *x*(*t*) is

A.11 $$ \tilde{x}(\omega) = {\int}_{-\frac{T}{2}}^{\frac{T}{2}} x(t) \exp (-i \omega t) dt. $$

To quantify the fluctuations about the endemic equilibrium or about a solution of a fast-slow system at some arbitrary time *t*, we note the following system of stochastic differential equations is equivalent to Eq. A.9,

$$\begin{array}{@{}rcl@{}} dx_{1} &=&a_{11} x_{1}(t)dt + a_{12} x_{2}(t)dt + \sqrt{D_{11}} d\tilde{W_{1}}(t) + \sqrt{D_{12}} d\tilde{W_{2}}(t)\\ dx_{2}&=& a_{21} x_{1}(t)dt + a_{22} x_{2}(t)dt + \sqrt{D_{21}} d\tilde{W_{1}}(t)+ \sqrt{D_{22}} d\tilde{W_{2}}(t),  \end{array} $$

where $d\tilde {W_{i}}(t)$ are independent Wiener processes. For notational convenience, we express these equations as

A.12 $$\begin{array}{@{}rcl@{}} \frac{dx_{1}}{dt} &=&a_{11} x_{1}(t) + a_{12} x_{2}(t) + {\Gamma}_{1}(t) \\ \frac{dx_{2}}{dt}&=&a_{21} x_{1}(t) + a_{22} x_{2}(t) + {\Gamma}_{2}(t),  \end{array} $$

where Γ_1_(*t*) and Γ_2_(*t*) represent white noise processes associated with the covariance matrix *D*. To analyze the system (A.12), we take its Fourier transform, leading to

A.13 $$\begin{array}{@{}rcl@{}} i \omega \tilde{x_{1}}(\omega) &=&a_{11} \tilde{x_{1}}(\omega) + a_{12} \tilde{x_{2}}(\omega) + \tilde{{\Gamma}_{1}}(\omega) \\ i \omega \tilde{x_{2}}(\omega)&=& a_{21}\tilde{x_{1}}(\omega) + a_{22} \tilde{x_{2}}(\omega) +\tilde{{\Gamma}_{2}}(\omega),  \end{array} $$

where $\tilde {x_{1}}(\omega )$, $\tilde {x_{2}}(\omega )$, $\tilde {{\Gamma }_{1}}(\omega )$, and $\tilde {{\Gamma }_{2}}(\omega )$ are the Fourier transforms of *x*_1_(*t*), *x*_2_(*t*), Γ_1_(*t*), and Γ_2_(*t*), respectively. We note that in Table 5, we have defined the *a*_*i**j*_ coefficients in terms of time. If the aim is to calculate summary statistics about an endemic equilibrium at time *t*, then these coefficients are constant values. If we calculate the statistics for the fluctuations about a solution of the fast-slow system at some arbitrary time *t*, we use the values of the *a*_*i**j*_ at that time *t*. This is reasonable because we assume that the control measure being taken is changing very slowly relative to the time scale of the evolution of infectious human hosts. Consequently, we assume these coefficients are constant relative to the time scale that the number of infectious hosts are changing over in an arbitrary finite time interval.

Since we are interested in fluctuations about the infectious human state, we solve (A.13) for the Fourier transform $\tilde {x_{1}}(\omega )$, obtaining

A.14 $$ \tilde{x_{1}}(\omega) = \frac{(a_{22}-i \omega ){\Gamma}_{1}(\omega)}{d-\omega^{2}+i T \omega}-\frac{a_{12}{\Gamma}_{2}(\omega)}{d-\omega^{2}+i T \omega},  $$

where *T* and *d* are the trace and determinant of the Jacobian matrix respectively, given in Table 5. Using (A.14), we can establish the power spectrum of the fluctuations $\langle |\tilde {x_{1}}(\omega )^{2}|\rangle $,

A.15 $$ S_{H}(\omega)=\frac{(\alpha_{H}+D_{11} \omega^{2})}{(\omega^{2}-d)^{2}+T^{2} \omega^{2}},  $$

where *α*_*H*_ is given in Table 5. The variance, autocorrelation and coefficient of variation of the fluctuations in *H*(*t*) around the endemic equilibrium may be obtained using Eq. A.15 through integration, (e.g., as in (Nisbet and Gurney 1982; O’Regan and Drake 2013)), see Table 4 for expressions.

## B Appendix

To establish that the endemic equilibrium of the Ross-Macdonald equations (system (1) in the main text) is always a stable node, and thus eigenvalues obtained from the Jacobian matrix

$$A=\left( \begin{array}{ll} a_{11} & a_{12} \\ a_{21} & a_{22} \end{array}\right) $$

evaluated at the endemic equilibrium are negative, real, and distinct, we need to establish that the trace of the Jacobian matrix tr(*A*) is negative, the determinant det(*A*) is strictly positive, and the radical in the eigenvalues can never be negative, i.e., (*t**r*(*A*))^2^−4 det(*A*) > 0. Entries of the Jacobian matrix are given in Table 5. Clearly, *t**r*(*A*) = *a*_11_ + *a*_22_=−*k**p**M*^∗^/*N*_*h*_−*μ*−*k**q**H*^∗^/*N*_*h*_−*δ* is strictly negative since *k*, *p*, *q*, *N*_*h*_, *μ*, *δ* > 0 and the endemic equilibrium (*H*^∗^, *M*^∗^) is strictly positive. Next, we need to establish that the determinant of *A* is strictly positive,

$$\begin{array}{@{}rcl@{}} a_{11} a_{22} - a_{12} a_{21} &=& \delta k p \frac{M^{\ast}}{N_{h}} + \mu k q \frac{H^{\ast}}{N_{h}}+ \mu \delta - k^{2} p q \frac{N_{m}}{N_{h}} \\ &&+ k^{2} p q \frac{M^{\ast}}{N_{h}} + k^{2} p q H^{\ast}\frac{N_{m}}{{N_{h}^{2}}}. \end{array} $$

Given that *R*_0_ = *k*^2^*p**q**N*_*m*_/*μ**δ**N*_*h*_, we can write this expression as

$$k p \frac{M^{\ast}}{N_{h}} (\delta + k q) + \frac{H^{\ast}k q} {N_{h}}\left( \mu + \frac{k p N_{m}}{N_{h}}\right) - \mu \delta (R_{0} - 1). $$

Rewriting *H*^∗^ and *M*^∗^ in terms of *R*_0_ and cancelling out terms yields the condition

$$\mu \delta (R_{0} - 1) > 0. $$

Since *μ* and *δ* are positive constants, then *R*_0_ > 1 is required for the endemic equilibrium to be stable. Finally, we need to establish that the radical in the eigenvalues can never be negative. The condition for a stable node is

B.1 $$ (a_{11}- a_{22})^{2} +4 a_{12} a_{21} > 0. $$

The condition is satisfied if either (i) *a*_12_, *a*_21_≥0 or (ii) *a*_12_, *a*_21_≤0. From the Jacobian matrix, *a*_12_ = *k**p*(*N*_*h*_−*H*^∗^)/*N*_*h*_ and *a*_21_ = *k**q*(*N*_*m*_−*M*^∗^)/*N*_*h*_. Since *k*, *p*, *q* and *N*_*h*_ are all strictly positive and the equilibrium susceptible human and mosquito host populations are always greater than zero, then condition (B.1) is always satisfied. Moreover, the node is not degenerate since the equilibrium susceptible human and mosquito host populations are strictly positive, and thus the eigenvalues are never equal.

## References

[CR1] Allen EJ, Allen LJS, Arciniega A, Greenwood PE (2008) Construction of equivalent stochastic differential equation models. Stochastic Analysis Applied

[CR2] Allen LJS (2003). An introduction to stochastic processes with applications to biology.

[CR3] Boettiger C, Hastings A (2012) Quantifying limits to detection of early warning for critical transitions. J R Soc Interface 9(75):2527–2539. doi:10.1098/rsif.2012.0125 10.1098/rsif.2012.0125 PMC3427498 22593100

[CR4] Breman JG, De Quadros CA, Dowdle WR, Foege WH, Henderson DA, John TJ, Levine MM (2011). The role of research in viral disease eradication and elimination programs: lessons for malaria eradication. PLoS Med.

[CR5] Chiyaka C, Tatem AJ, Cohen JM, Gething PW, Johnston G, Gosling R, Laxminarayan R, Hay SI, Smith DL (2013). Infectious disease. The stability of malaria elimination. Science.

[CR6] Cohen JM, Moonen B, Snow RW, Smith DL (2010). How absolute is zero? An evaluation of historical and current definitions of malaria elimination. Malar J.

[CR7] Cohen JM, Smith DL, Cotter C, Ward A, Yamey G, Sabot OJ, Moonen B (2012). Malaria resurgence: a systematic review and assessment of its causes. Malar J.

[CR8] Dakos V, Scheffer M, Van Nes EH, Brovkin V, Petoukhov V, Held H (2008). Slowing down as an early warning signal for abrupt climate change. Proc Natl Acad Sci USA.

[CR9] Dakos V, van Nes EH, D’Odorico P, Scheffer M (2012). Robustness of variance and autocorrelation as indicators of critical slowing down. Ecology.

[CR10] Ferrari MJ, Bjørnstad ON, Dobson AP (2005). Estimation and inference of R0 of an infectious pathogen by a removal method. Math Biosci.

[CR11] Giardina F, Kasasa S, Sié A, Utzinger J, Tanner M, Vounatsou P (2014). Effects of vector-control interventions on changes in risk of malaria parasitaemia in sub-Saharan Africa: a spatial and temporal analysis. Lancet Glob Health.

[CR12] Goodman CA, Coleman PG, Mills AJ (1999). Cost-effectiveness of malaria control in sub-Saharan Africa. Lancet.

[CR13] Gore-Langton GR, Alenwi N, Mungai J, Erupe NI, Eves K, Kimwana FN, Soti D, Akhwale W, Hassan FA, Juma E, Allan R (2015). Patient adherence to prescribed artemisinin-based combination therapy in Garissa County, Kenya, after three years of health care in a conflict setting. Malar J.

[CR14] Gratz NG (1999). Emerging and Resurging Vector-Borne Diseases. Ann Rev Entomol.

[CR15] Gubler DJ (1998) Resurgent vector-borne diseases as a global health problem. Emerg Infect Dis 4(3):442–50. doi:10.3201/eid0403.980326 10.3201/eid0403.980326 PMC2640300 9716967

[CR16] Gubler DJ (2010) The global threat of emergent/re-emergent vector-borne diseases. In: Atkinson PW (ed) Vector Biology, Ecology and Control, pp 39–63

[CR17] Hollingsworth TD, Pulliam JR, Funk S, Truscott JE, Isham V, Lloyd AL (2014) Seven challenges for modelling indirect transmission: Vector-borne diseases, macroparasites and neglected tropical diseases. Epidemics doi:10.1016/j.epidem.2014.08.007 10.1016/j.epidem.2014.08.007 PMC4383804 25843376

[CR18] Hopkins DR (2013). Disease Eradication. N Engl J Med.

[CR19] Institute of Medicine (US) Forum on Microbial Threats (2008) Vector-Borne Disease Emergence and Resurgence

[CR20] Keeling MJ, Rohani P (2008). Modeling infectious diseases in humans and animals.

[CR21] Klepac P, Metcalf CJE, McLean AR, Hampson K (2013). Towards the endgame and beyond: complexities and challenges for the elimination of infectious diseases. Phil Trans R Soc B.

[CR22] Kuehn C (2011). A mathematical framework for critical transitions: bifurcations, fast-slow systems and stochastic dynamics. Phys D.

[CR23] Lawpoolsri S, Klein EY, Singhasivanon P, Yimsamran S, Thanyavanich N, Maneeboonyang W, Hungerford LL, Maguire JH, Smith DL (2009). Optimally timing primaquine treatment to reduce Plasmodium falciparum transmission in low endemicity Thai-Myanmar border populations. Malar J.

[CR24] Mackenzie JS, Gubler DJ, Petersen LR (2004). Emerging flaviviruses: the spread and resurgence of Japanese encephalitis, West Nile and dengue viruses. Nat Med.

[CR25] Nisbet RM, Gurney WSC (1982). Modelling fluctuating populations.

[CR26] Noor AM, Amin AA, Akhwale WS, Snow RW (2007). Increasing coverage and decreasing inequity in insecticide-treated bed net use among rural Kenyan children. PLoS Med.

[CR27] Nyarango PM, Gebremeskel T, Mebrahtu G, Mufunda J, Abdulmumini U, Ogbamariam A, Kosia A, Gebremichael A, Gunawardena D, Ghebrat Y, Okbaldet Y (2006). A steep decline of malaria morbidity and mortality trends in Eritrea between 2000 and 2004: the effect of combination of control methods. Malar J.

[CR28] O’Regan SM, Drake JM (2013). Theory of early warning signals of disease emergence and leading indicators of elimination. Theor Ecol.

[CR29] Paaijmans KP, Read AF, Thomas MB (2009) Understanding the link between malaria risk and climate. Proceedings National Academic Science USA pp 13,844–13,849 doi:10.1073/pnas.0903423106 10.1073/pnas.0903423106 PMC2720408 19666598

[CR30] Paaijmans KP, Cator LJ, Thomas MB (2013). Temperature-dependent pre-bloodmeal period and temperature-driven asynchrony between parasite development and mosquito biting rate reduce malaria transmission intensity. PloS One.

[CR31] Perkins TA, Scott TW, Le Menach A, Smith DL (2013). Heterogeneity, mixing, and the spatial scales of mosquito-borne pathogen transmission. PLoS Comput Biol.

[CR32] Read AF, Lynch PA, Thomas MB (2009). How to make evolution-proof insecticides for malaria control. PLoS Biol.

[CR33] Reiner RC, Perkins TA, Barker CM, Niu T, Chaves LF, Ellis AM, George DB, Menach AL, Pulliam JRC, Bisanzio D, Buckee C, Chiyaka C, Cummings DAT, Garcia AJ, Gatton ML, Gething PW, Hartley DM, Johnston G, Klein EY, Michael E, Lindsay SW, Lloyd AL, Pigott DM, Reisen WK, Ruktanonchai N, Singh BK, Tatem AJ, Kitron U, Hay SI, Scott TW, Smith DL (2013). A systematic review of mathematical models of mosquito-borne pathogen transmission: 1970–2010. J R Soc Interface.

[CR34] Rogers DJ, Randolph SE, Snow RW, Hay SI (2002). Satellite imagery in the study and forecast of malaria. Nature.

[CR35] Scheffer M, Bascompte J, Brock WA, Brovkin V, Carpenter SR, Dakos V, Held H, Van Nes EH, Rietkerk M, Sugihara G (2009). Early-warning signals for critical transitions. Nature.

[CR36] Smith DL, McKenzie FE (2004). Statics and dynamics of malaria infection in Anopheles mosquitoes. Malar J.

[CR37] Smith DL, Battle KE, Hay SI, Barker CM, Scott TW, McKenzie FE (2012). Ross, Macdonald, and a theory for the dynamics and control of mosquito-transmitted pathogens. PLoS Pathog.

[CR38] Strogatz SH (1994). Nonlinear dynamics and chaos with applications to physics, biology, chemistry and engineering.

[CR39] Tusting LS, Thwing J, Sinclair D, Fillinger U, Gimnig J, Bonner KE, Bottomley C, Lindsay SW (2013). Mosquito larval source management for controlling malaria. Cochrane Database Syst Rev.

[CR40] World Health Organization (2013) World health report: research for universal health coverage. Technical Report

[CR41] World Health Organization (2014) Vector-borne diseases. fact sheet No. 387

